# 2082 Profile of pediatric potentially avoidable transfers

**DOI:** 10.1017/cts.2018.301

**Published:** 2018-11-21

**Authors:** Jennifer Rosenthal, James Marcin, Monica Lieng, Patrick Romano

**Affiliations:** University of California, Davis, CA, USA

## Abstract

OBJECTIVES/SPECIFIC AIMS: While hospital-hospital transfers of pediatric patients is often necessary, some pediatric transfers are potentially avoidable. Pediatric potentially avoidable transfers (PAT) represent a process with high costs and safety risks but few, if any, benefits. To better understand this issue, we described pediatric inter-facility transfers with early discharges. METHODS/STUDY POPULATION: We conducted a descriptive study using electronic medical record data at a single-center over a 12-month period to examine characteristics of pediatric patients with a transfer admission source and early discharge. Among patients with early discharges, we performed descriptive statistics for PAT defined as patient transfers with a discharge home within 24 hours without receiving any specialized tests, interventions, consultations, or diagnoses. RESULTS/ANTICIPATED RESULTS: Of the 2414 pediatric transfers 31.2% were discharged home within 24 hours. Among transferred patients with early discharges, 348 patients (14.4% of total patient transfers) received no specialized tests, interventions, consultations, or diagnoses. Direct admissions were categorized as PAT 2.2-fold more frequently than transfers arriving to the emergency department. Among transferred direct admissions, PAT proportions to the neonatal intensive care unit (ICU), pediatric ICU, and non-ICU were 5.8%, 17.4%, and 27.3%, respectively. Respiratory infections, asthma, and fractures were the most common PAT diagnoses. DISCUSSION/SIGNIFICANCE OF IMPACT: Early discharges and PAT are relatively common among transferred pediatric patients. Further studies are needed to identify the etiologies and clinical impacts of PAT, with a focus on direct admissions given the high frequency of PAT among direct admissions to both the pediatric ICU and non-ICU.

